# Casimir Force in Layered Materials and Control of the Stable Equilibrium

**DOI:** 10.1021/acs.jpclett.5c03101

**Published:** 2025-11-25

**Authors:** Connor Williamson, Elena Besley

**Affiliations:** School of Chemistry, 6123 University of Nottingham, Nottingham NG7 2RD, United Kingdom

## Abstract

Quantum interactions between nearly touching neutral interfaces lead to a measurable Casimir force, which is often assumed to be attractive. Our comprehensive computational analysis of atomic force microscopy and surface force apparatus measurements of the Casimir force between layered materials in solvents confirms that the Casimir interactions can give rise to a stable equilibrium. Given the excellent agreement with experiments for a range of materials, we extend the formalism to multiple-layer interfaces and investigate the effects of solvent and compositional changes on the Casimir equilibrium. We explain how the Casimir equilibrium can be tuned precisely and show that the scaling of the equilibrium distance with the thickness of the top layer is a controllable, solvent-dependent parameter. These findings have significant implications for the self-assembly of layered materials and design of future quantum entrapment experiments.

## Graphical abstract

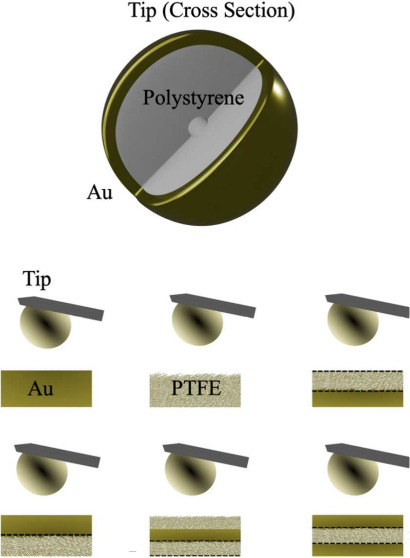

The Casimir effect, a visionary theoretical prediction^1^ that two uncharged dielectric or conducting interfaces experience attraction at very close separations, is purely quantum mechanical in nature. It stems from the presence of fluctuating electromagnetic fields on either side of a nanometer-size cavity created between the interfaces, which exist even in a perfect vacuum and can be suppressed or enhanced inside the cavity. This creates a slight difference in the energy densities inside and outside the cavity. In the case of mirror-reflective geometries and if the interfaces are made of the same material, a net attractive force is established which is independent of the shape of the interacting bodies and their dielectric properties.^2^ However, this is not a universal rule, and repulsion between interfaces made of the same material can be also achieved, as predicted by Levin et al.^3^ for the case of the interaction between a needle and a plate. Due to the lack of sophisticated instrumentation capable of measuring these subtle changes, the Casimir effect has remained experimentally elusive for almost 50 years, drawing little interest outside of the theoretical community. After the first unambiguous measurements by Lamoreaux^4^ of the Casimir force between a flat metal plate and a sphere, experimental evaluations of the attractive Casimir force have been realized in a range of geometries.^5–8^

The Lifshitz theory of van der Waals forces^9,10^ states that the Casimir force can change from attractive to repulsive through a suitable choice of interacting materials immersed in a fluid. First experimental evidence confirming the general Lifshitz theory was reported by Munday et al.^11^ They showed that repulsive Casimir interactions can be realized and measured if the walls of the cavity are composed of materials with different dielectric response functions, ϵ_1_(*iξ*) and ϵ_2_(*iξ*), such that the dielectric response function of the medium separating the walls satisfies the following criterion,

1 $$\epsilon_{1}\left(i\xi \right)>\epsilon_{m}\left(i\xi \right)>\epsilon_{2}\left(i\xi \right)$$

over the relevant range of Matsubara frequencies. Although the measured repulsive force was found to be weaker than the attractive force established between two gold surfaces in the same medium, the magnitude of both forces increases with decreasing surface separation, in complete agreement with the Lifshitz theory. The existence of repulsive Casimir–Lifshitz force has been further confirmed, and several interesting experimental setups studied these repulsive interactions^3,12–16^ and optical properties of the interfaces.^17^

An explosive potential of the Casimir effect has become apparent with the rise of research in plasmonics and metamaterials, where the Casimir force can be manipulated and investigated at levels of accuracy and versatility inaccessible to conventional methods.^8,18–22^ Nanopatterning of metallic surfaces has opened up further opportunities for exploring novel phases that emerge at the interfaces affected by the Casimir force.^23–25^ The developments of micro- and nano-electromechanical systems could probe not only repulsive but also zero Casimir forces (equilibrium).^6,26–30^ Many important theoretical studies have also explored how the Casimir equilibrium can be tuned in various layered systems using phase change materials, electrolyte solutions, and different material compositions.^31–38^

In the era of miniaturization of integrated electronic devices, the importance of unlocking the full potential of the Casimir force cannot be underestimated, as it can be exploited in delivering room-temperature, low-cost precision quantum measurements conducted in the deep submicron regime.^39^ However, progress depends on our ability to control and fine-tune the attractive and repulsive Casimir interactions, leading ultimately to a high-precision manipulation of the Casimir equilibrium. For example, the switch between attraction and repulsion has been realized in external magnetic fields in ferrofluids.^40,41^ While the nature of the Casimir force can be controlled by external perturbations, the force–distance profile is largely defined by the materials involved. Experiments by Zhao et al.^42^ achieved a stable Casimir equilibrium for a gold nanoplate suspended in ethanol above a Teflon-coated gold surface, thus demonstrating quantum entrapment of the nanoplate at a fixed height above the surface. If the permittivity relation ([Disp-formula eq1]) is satisfied over the relevant frequency range, then these two interfaces repel at all separation distances, as also demonstrated by the measurements of the repulsive Casimir force between Teflon and gold surfaces in cyclohexane.^12,14^

In this paper, we present quantitatively accurate predictions of the Casimir effect in layered materials of increasing complexity, building up from simple single-layer interfaces to double-layer structures and, finally, to multiple-layer materials of arbitrary composition and thickness. These predictions are in excellent agreement with the existing experimental data for a range of complex systems. We further show how the Casimir force, equilibrium separation, and quantum entrapment can be controlled by a simple manipulation of the order of the layers and medium. This brings us closer to achieving a full understanding and control of this interesting and indispensable phenomenon.

The Casimir–Polder formalism^43^ is a generalization of the description of van der Waals force in the vicinity of a macroscopic interface which includes retardation due to the finite speed of light. Lifshitz theory^9,10^ agrees with van der Waals theory in the short separation limit where retardation has minimal effects on the Casimir force. The experimentally measured force typically includes the Casimir and electrostatic contributions, and the latter is considered to be a background effect which needs to be excluded from the measurements.^44^ To minimize the electrostatic force, nearly all experiments have been performed with well-conducting materials. Following Parsegian,^45^ we represent the interacting surfaces *A* and *B* as two half-spaces separated by a medium, *m*, with the dielectric function, ϵ_*m*_(*iξ*), and thickness, l (Figure S1 in the Supporting Information). The Casimir interaction free energy (per unit area) can be then defined as the summation of the free energies of the allowed fluctuation modes at the interface (surface modes),

2 $$G\left(l,i\xi_{n}\right)=\frac{kT}{2\pi c^{2}}\overset{\infty }{\underset{n=0}{\sum }}\epsilon_{m}\left(i\xi_{n}\right)\xi_{n}^{2}\int_{1}^{\infty }p\ln\left[D\left(l,i\xi_{n}\right)\right]\;dp$$

where *k* is the Boltzmann constant, *T* is the temperature, and *c* is the speed of light. The dielectric function of the material, ϵ­(*iξ*_*n*_), is defined at the relevant Matsubara frequencies,

$$\xi_{n}=\frac{2\pi nkT}{\hbar }$$

where *ℏ* is the reduced Planck constant. As the summation in eq 2 is taken over positive values of *n*, the *n* = 0 term needs to be multiplied by 1/2 to avoid double counting of this term. For each complex frequency *iξ*_*n*_, the function *D*( l , *iξ*_*n*_) includes the dielectric properties of the boundaries between surface *A* and the medium, *A*|*m*, and surface *B* and the medium, *B*|*m*, as

3 $$D\left(l,i\xi_{n}\right)=\left(1-\Delta_{A|m}\left(i\xi_{n}\right)\Delta_{B|m}\left(i\xi_{n}\right)\;e^{-2\rho_{m}l}\right)$$

$$\rho_{m}=\frac{\sqrt{\epsilon_{m}\left(i\xi_{n}\right)}\xi_{n}}{c}p$$

where 1 ≤ *p* < *∞*. Δ­(*iξ*_*n*_) varies depending on the geometry of the interface, and in the simplest case of a single composition half-space, it takes the following form for the surface *A*:

4 $$\Delta_{A|m}=\frac{s_{A}\epsilon_{m}-s_{m}\epsilon_{A}}{s_{A}\epsilon_{m}+s_{m}\epsilon_{A}}$$

$$s_{A}={\left(p^{2}-1+\left(\epsilon_{A}/\epsilon_{m}\right)\right)}^{1/2},,s_{m}=p$$

where ϵ_*A*_ = ϵ_*A*_(*iξ*_*n*_) is the dielectric function of the surface *A*, and *s*_*A*_ is the corresponding component of the radial wave vector at the same frequency. A separate expression for Δ_*B*|*m*_ is derived by replacing *A* for *B* in eqs 4.

As all materials studied in this work are nonmagnetic, we neglect the magnetic dependence of the Casimir energy and assume the relative magnetic permeability of the materials to be unity (μ = 1), which is commonly accepted in the literature.^11,12,42,46^ The dielectric permittivity of most materials is not sensitive to external magnetic fields. However, in the Lifshitz theory,^9,10^ the Casimir force is shown to be also affected by the magnetic permeability of the materials, and this effect is particularly prominent in ferrofluids, as discussed, for example, by Zhang et al.^41^ These magnetic materials open up additional opportunities to tune the Casimir force by using external magnetic fields. A rigorous procedure for calculating the Casimir attraction with arbitrary magnetic and dielectric properties can be found in Ellingsen’s work.^47^

Some accurate oscillator models^45,48^ calculate ϵ­(*iξ*) directly, where the dielectric function takes the general form of

5 $$\epsilon \left(i\xi \right)=1+\underset{l}{\sum }\frac{d_{l}}{1+\xi \tau_{l}}+\underset{k}{\sum }\frac{f_{k}}{\omega_{k}^{2}+g_{k}\xi +\xi^{2}}$$

The oscillator form ([Disp-formula eq5]) considers a damped harmonic oscillator model, where the first summation (over *l*) describes the Debye dipolar relaxation; τ_*l*_ is the relaxation time, and *d*_*l*_ is analogous to the oscillator strength. Higher order frequency terms are accounted for in the second summation (over *k*) in a damped oscillator form. The constants *f*_*k*_ and *g*_*k*_ in eq 5 have been determined for many well-studied materials and have shown excellent agreement with the literature.^45,48^ Alternative oscillator models include^12^

6 $$\epsilon \left(i\xi \right)=1+\underset{l}{\sum }\frac{C_{l}}{1+{\left(\xi /\omega_{l}\right)}^{2}}$$

where ω_*l*_ is the resonance frequency and *C*_*l*_ is the oscillator strength. In this study, oscillator models ([Disp-formula eq5]) and ([Disp-formula eq6]) have been used along with the recently optimized oscillators by Gudarzi and Aboutalebi,^49^ which have been shown to accurately reproduce experimental results.

To study the Casimir effect in materials with multiple layers, Sernelius^50^ focused on calculating the Casimir effects in systems containing two-dimensional (2D) layers such as graphene and 2D electron gases. Tomaš^51^ extended the Lifshitz formalism to account for the presence of layered medium. Here, we calculate the Casimir effect in materials with multiple layers of arbitrary thickness. If a layer *A*_1_ with thickness *a*_1_ is added to the interface described by the half-space *A*, then Δ_*A*|*m*_ in eq 3 is transformed to ${\mathrm{\Delta ̅}}_{A|m}$ as follows:

7 $${\mathrm{\Delta ̅}}_{A|m}=\frac{\Delta_{A|A_{1}}\;e^{-2\rho_{A_{1}}a_{1}}+\Delta_{A_{1}|m}}{1+\Delta_{A_{1}|m}\Delta_{A|A_{1}}\;e^{-2\rho_{A_{1}}a_{1}}}$$

where

$$\rho_{A_{1}}={\left(\rho_{m}^{2}+{\left(\xi_{n}/c\right)}^{2}\left(\epsilon_{A_{1}}-\epsilon_{m}\right)\right)}^{1/2}$$

Adding another layer, *A*_2_, with thickness *a*_2_ (Figure S1 in the Supporting Information) requires $\Delta_{A|A_{1}}$ in eq 7 to be replaced with

8 $$\frac{\Delta_{A|A_{2}}\;e^{-2\rho_{A_{2}}a_{2}}+\Delta_{A_{2}|A_{1}}}{1+\Delta_{A|A_{2}}\Delta_{A_{2}|A_{1}}\;e^{-2\rho_{A_{2}}a_{2}}}$$

The subsequent addition of layers proceeds by induction so that with each added layer the functions $\Delta_{A|A_{i}}$ get transformed as

9 $$\frac{\Delta_{A|A_{i+1}}\;e^{-2\rho_{A_{i+1}}a_{i+1}}+\Delta_{A_{i+1}|A_{i}}}{1+\Delta_{A|A_{i+1}}\Delta_{A_{i+1}|A_{i}}\;e^{-2\rho_{A_{i+1}}a_{i+1}}}$$

Once again, similar expressions need to be derived for the surface *B*.

The form ([Disp-formula eq2]) for the Casimir interaction free energy is convenient for obtaining the Casimir force numerically as the differential with respect to the cavity size, l , following *F*( l ) = −d*G*( l )/d l . In earlier experimental works,^11,12,42,45,46,48,49^ the Derjaguin approximation was widely used to relate the Casimir energy and force. In atomic force microscopy (AFM) and surface force apparatus (SFA) experiments, the Casimir force is often scaled to reduce the problem to a sphere–plane solution (in AFM) or a perpendicular cylinder–cylinder solution (in SFA) as follows:^45,46,48^

10 $$F_{\mathrm{sphere-plane}}\left(l\right)=F_{cyl⊥cyl}\left(l\right)=2\pi R_{eff}G\left(l\right)$$

Here, *R*_*eff*_ is the radius of the sphere or cylinder, or $\sqrt{R_{1}R_{2}}$ if the cylinders are of different sizes. The Derjaguin approximation ([Disp-formula eq10]) can be used when the radius of curvature is much larger than the separation distance, namely *R*_*eff*_ ≫ l . In this work, however, we also use numerical derivative of the free energy to ensure a high precision in locating the equilibrium, defined by *F*( l ) = 0.

In the following, we investigate the changing nature of the Casimir effect in layered materials and demonstrate how control of the Casimir equilibrium can be achieved by making simple compositional changes in the layered materials and the medium. All calculations were performed using MATLAB and associated solvers.^52^ The absolute error of 10^–6^ in the global adaptive quadrature-based numerical integration routines was deemed appropriate through benchmarking the convergence of the Casimir energy (eq 2). The value *n* of the surface modes of the fluctuating electric field was set to 2^12^ to achieve a convergence of the total energy to within 1% when compared to a calculation with a higher number of modes (*n* = 2^13^).

We first consider the case of single-layer interfaces reported by Munday et al.,^11^ where AFM was used to demonstrate the effect of the dielectric function of the interacting surfaces and medium on the overall Casimir interaction. They presented two examples: attractive Casimir force acting between two gold interfaces (Au Casimir force tip–Au surface) and repulsive Casimir force between gold and silica interfaces (Au Casimir force tip–SiO_2_ surface), both measured in bromobenzene (see Figure 1). We calculated the interaction energy in these two distinct setups using the oscillator model described by van Zwol et al.^12^ The Derjaguin approximation was then applied to evaluate the Casimir force. Figure 1 shows excellent agreement, within the experimental error, between our computational predictions and the experiments of Munday et al.^11^ down to the surface separation distances of 20 nm. The small deviation from experiment at separations below 30 nm may be attributed to factors like surface roughness and purity and the limitations of the employed optical models at very short separations.^53^

**Figure 1:**
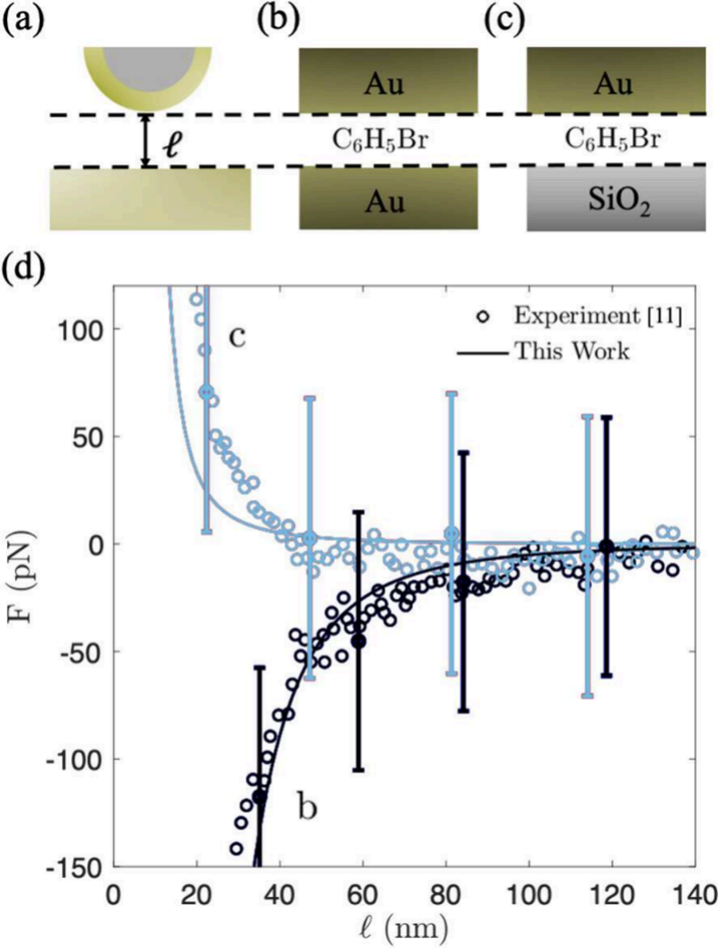
Casimir force acting between single-layer interfaces in bromobenzene: illustration of AFM experiments (a); computational setup (planar surfaces) for Au–Au interface (b) and Au–SiO_2_ interface (c). The Casimir force as a function of the separation distance (d) showing the results of the experimental AFM measurements^11^ (open circles) and calculated in this work (solid line) using eq 2 and the Derjaguin approximation, eq 10.

Previously, Ederth^48^ also reported a strong attractive Casimir force (of the order of μN per meter) measured by SFA in air between two identical gold surfaces with a more complex double-layer structure containing a hydrocarbon layer on top of gold. We calculated the Casimir force acting between double-layer Au–hydrocarbon interfaces in air (shown in Figure S3 in the Supporting Information) and found it to be in excellent agreement with the measurements of Ederth.^48^ In addition, Ederth also considered the effect of the electrostatic forces between gold surfaces covered with a hydrocarbon layer caused by residual potential differences. He concluded that the contribution to the total force from the electrostatic interactions is negligible at both long and short separation distances, even in the presence of a thick water layer, which would accumulate maximum charge. In this case, the total force acting on the SFA tip changes only by 1%.^48^

The presented computational setup also makes it possible to consider more delicate scenarios where the attractive and repulsive Casimir forces are kept in balance to maintain a stable equilibrium. The Casimir equilibrium has been recently observed by Zhao et al.,^42^ where a thin, μm-wide gold flake suspended in ethanol (EtOH) was trapped at a fixed height above a double-layer surface composed of gold and a thin overlayer of polytetrafluoro­ethylene (PTFE). In this case, the equilibrium (or quantum trapping) is maintained by the competing short-range repulsive forces between the gold flake and PTFE and the long-range attraction between the gold surfaces. Equation 2 and Figure 2 highlight that the Casimir interaction (repulsion, attraction, or equilibrium) depends on the frequency regime over which the permittivity relation ([Disp-formula eq1]) is satisfied. At different values of imaginary frequency, the value and order of the complex dielectric function can change, particularly at the lower frequencies, which contribute more significantly to the overall Casimir interaction energy than the higher frequencies.

**Figure 2:**
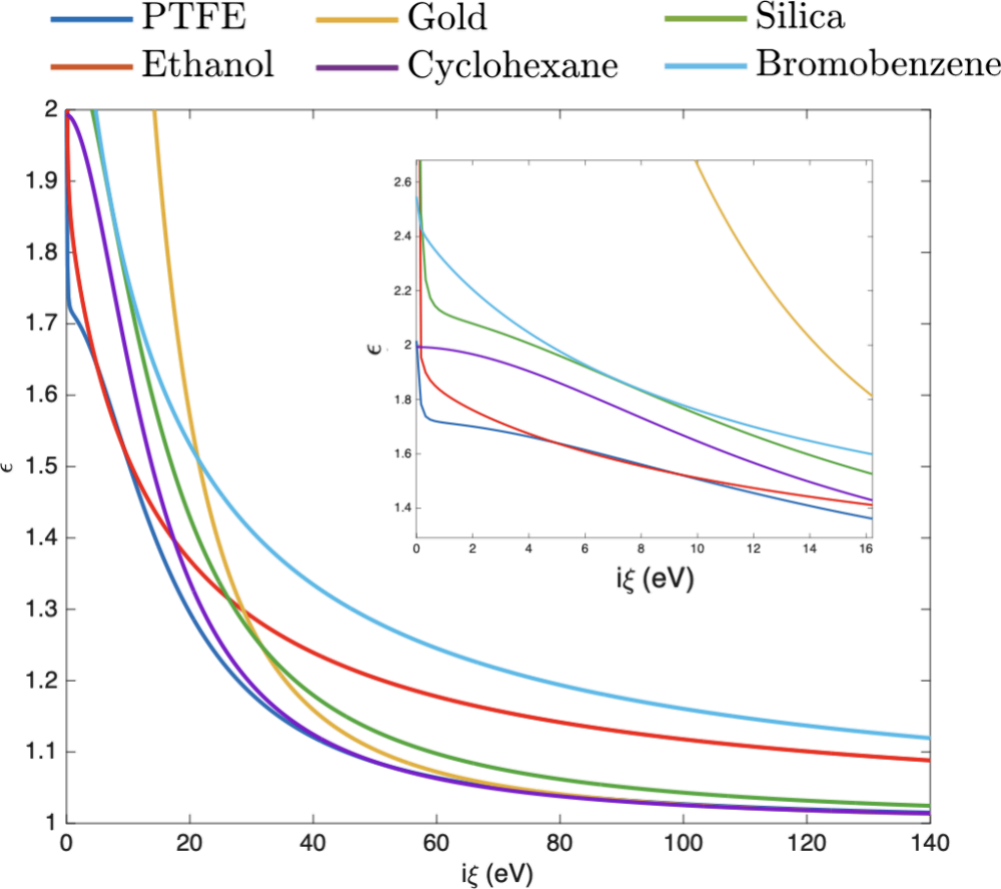
Dependence of the dielectric function on imaginary frequency for materials and solvents studied in this work.^12^

The double-layer PTFE–Au interface has been also used to investigate the measured equilibrium trapping distance of the gold flake above the interface while varying the thicknesses of the PTFE overlayer. A linear relationship was established to show that in ethanol the equilibrium trapping distance remains approximately half the PTFE thickness.^42^ Our calculations (Figure 3) confirm the AFM and Fabry–Perot experiments^42^ not only for the presence of the stable equilibrium but also for the observed relationship between the equilibrium trapping distance and thickness of the PTFE layer. We also show that, in such an experimental setup, if the PTFE layer is thicker than 10 nm, the equilibrium trapping distance would be greater in cyclohexane. For example, for a 50 nm PTFE layer (green curve in Figure 3), the equilibrium separation between the Au flake and double-layer PTFE–Au interface is l _*eq*_ = 23 nm in ethanol and it is l _*eq*_ = 46 nm in cyclohexane, while the strength of the interaction at the trapping distance is tripled in ethanol. It is interesting to note that, if the PTFE layer is only 10 nm (blue curve in Figure 3), the equilibrium will be established at l _*eq*_ = 6 nm in both solvents. The rate of increase of the equilibrium separation with thickness of the PTFE layer doubles in cyclohexane, as indicated by the gradient of the straight lines in Figure 3 of 0.98 (±0.014) for cyclohexane (solid line) and 0.49 (±0.047) for ethanol (dashed line). This conclusion is indirectly supported by the observations of Van Zwol and Palasantzas^12^ that in cyclohexane the repulsion between gold and PTFE interfaces is doubled compared to that in ethanol.

**Figure 3:**
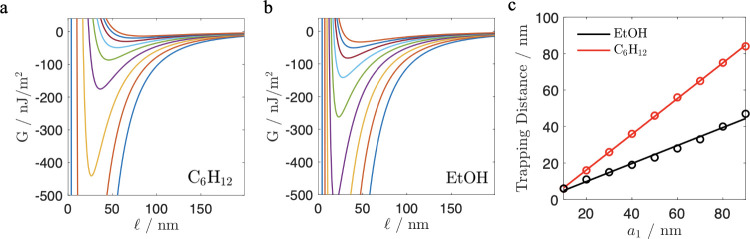
Casimir interaction free energy, per unit area, as a function of the separation, l , between Au surface and a PTFE–Au double layer in cyclohexane (a) and ethanol (b). The thickness of the top PTFE layer is 10 nm (blue), 20 nm (red), 30 nm (yellow), 40 nm (purple), 50 nm (green), 60 nm (light blue), 70 nm (maroon), 80 nm (dark blue), and 90 nm (orange). The minimum in the interaction energy curve, defined as the trapping distance, is shown in (c) as a function of the thickness, *a*_1_, of the PTFE overlayer for ethanol (black) and cyclohexane (red). The oscillator models are taken from ref 49.

A general overview of our computational predictions for the Casimir interaction energy, *G*, as a function of the separation distance, l , between the gold flake and a range of other interfaces is presented in Figure 4, where we continue to compare two solvents, ethanol and cyclohexane. Figure 4 contains a mixture of previously reported experimental setups (e.g., cases (a),^16^ (b),^12^ and (c)^42^) and other layered gold/PTFE layered materials which we predict could enable the manipulation of the position and strength of the Casimir equilibrium with some straightforward modifications to experiment. We show that, in the experimental setup used by Zhao et al.^42^ (case (c), dashed line), where the top PTFE overlayer is 60 nm thick, a stable equilibrium in ethanol is formed at l _*eq*_ = 28 nm. The interaction energy in this case is not affected by the number of layers at the interface as long as PTFE remains on top. For example, the interaction energy between the gold surface/flake (not shown in Figure 4) and the double-layer interface studied by Zhao et al.^42^ (case (c)) is the same as for the triple PTFE–Au–PTFE layer (case (e)) in both solvents, and it is independent of the thickness of the gold layer. This is because, at the trapping distances shown in Figure 4, the Casimir interaction is dominated by the dielectric properties of the top layer. When the top layer (PTFE, in this case) is sufficiently thick in comparison to the separation distance (e.g., 60 nm in Figure 4), the electromagnetic field is strongly screened at such separations, making the interaction largely insensitive to the composition of deeper layers and causing the dominant repulsion. We therefore conclude that going beyond the double-layer setup does not hold additional benefits in terms of control and manipulation of the Casimir equilibrium. Also, the intermediate gold layers can be made very thin without compromising the quality of the established equilibrium.

**Figure 4:**
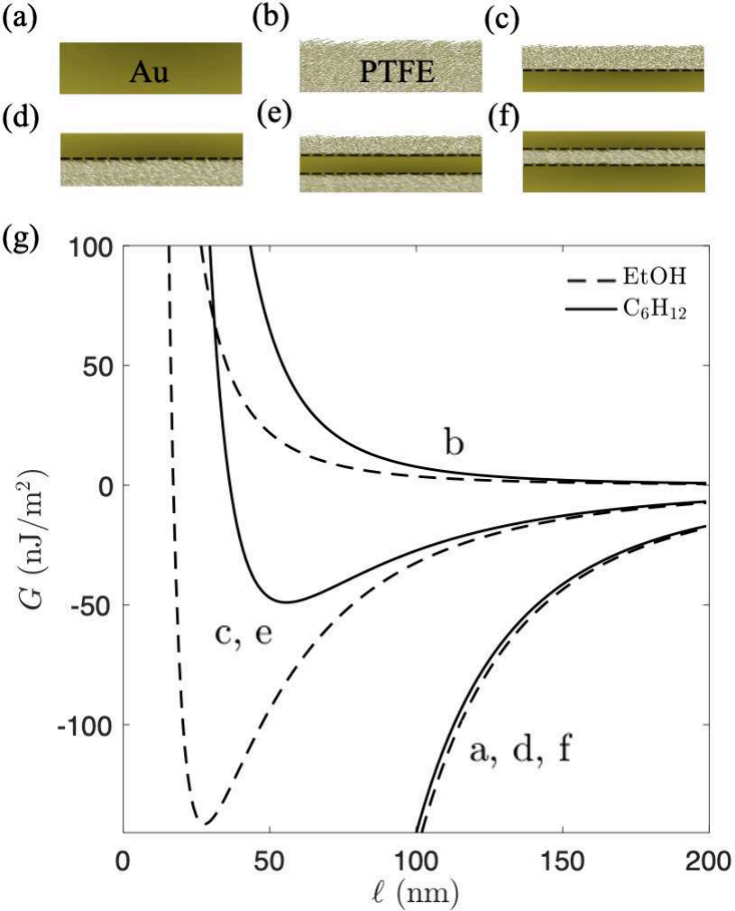
Casimir interaction free energy, per unit area, as a function of the separation distance between Au surface (not shown here) and a range of interfaces (a)–(f), in ethanol (dashed line) and cyclohexane (solid line). Note that the Au surface approaches the interfaces shown in (a)–(f) from the top. The dashed lines indicate the interface between different materials. The following thicknesses of the layers are assumed: (c) *a*_1_ = 60 nm; (d) *a*_1_ = 10 nm; (e) *a*_1_ = 60 nm and *a*_2_ = 90 nm; (f) *a*_1_ = 70 nm and *a*_2_ = 90 nm. The oscillator models are taken from ref 49.

Changing solvent, however, would significantly alter not only the equilibrium separation distance but also its strength. For example, for the experimental setup used by Zhao et al.^42^ (case (c)), the Casimir interaction energy at the equilibrium is about 3 times weaker in cyclohexane than in ethanol. Note that pure PTFE bulk repels the gold surface at all separation distances (case (b)). Furthermore, if PTFE and gold layers were swapped so that the gold layer was on top of a double- or triple-layer material (cases (d) and (f) in Figure 4), only attractive interactions could be established, and they would be identical to the case of two pure gold surfaces (case (a)). Once again, despite the layer thickness being explicitly present in the formalism, the strength of the Casimir attraction in the case of gold-terminated interfaces does not change with the thickness of the top layer in the case of gold–solvent–gold systems. This statement is valid for the layer thickness greater than the electromagnetic skin depth, which is typically of the order of tens of nm for the relevant frequencies.^54^ For layers thinner than 10 nm, gold becomes partially transparent to the fields, and the underlying PTFE begins to contribute a repulsive component to the force. This would require an alternative oscillator model to describe the dielectric properties of the constituent layers.

Given the great difference in the values of the dielectric constant of ethanol (ϵ = 24.3) and cyclohexane (ϵ = 2.02), it is encouraging to see that a strong, stable Casimir equilibrium and quantum trapping can be established in a variety of solvents. Further discussion of the solvent effect can be found in the Supporting Information. While the PTFE–gold interaction is largely affected by changing the medium, attraction between gold-terminated interfaces remains almost the same in cyclohexane and in ethanol (cases (a), (d), and (f) in Figure 4). This can be attributed to the much greater value of the dielectric function of gold compared to those of PTFE and the solvents, which makes the gold–gold interaction dominant in any medium.

In conclusion, this systematic quantitative study of the Casimir effect in layered materials builds upon previous experimental and theoretical works and extends our fundamental understanding of this complex quantum mechanical phenomenon. We explain how the Casimir equilibrium can be tuned and show a controllable, solvent-dependent scaling of the equilibrium distance with the thickness of the top layer. We establish experimental conditions in which the Casimir equilibrium can be achieved and demonstrate that, for the studied systems, a thin overlayer of PTFE is critical in the formation of the stable equilibrium, while adding subsequent layers has a negligible effect. We also show that gold-terminated surfaces experience purely attractive forces for any gold layer thicker than the electromagnetic skin depth (approximately 10 nm). These findings have significant implications on self-assembly of layered materials and design of future quantum entrapment experiments, given various interesting and experimentally viable layered interfaces.^55^

## Supplementary Material



## Notes

Schematic of the layered materials used; calculations of the dielectric functions for solvent mixtures; test results for the Casimir force between double-layer Au–hydrocarbon in air (PDF)
