# Supplementary material for: Casimir Force in Layered Materials and Control of the Stable Equilibrium
Source: J Phys Chem Lett. 2025 Nov 25;16(48):12470–6. doi: 10.1021/acs.jpclett.5c03101 (PMC12683632; doi:10.1021/acs.jpclett.5c03101)
Supplement: Supplementary file 1 [file jz5c03101_si_001.pdf]

Supporting Information for Publication. Casimir Force in Layered Materials and Control of the Stable Equilibrium

Connor Williamson and Elena Besley*

School of Chemistry, University of Nottingham, Nottingham NG7 2RD, United Kingdom

E-mail: Elena.Besley@nottingham.ac.uk

A schematic of the layered materials used in the computations are shown in Figure S 1.

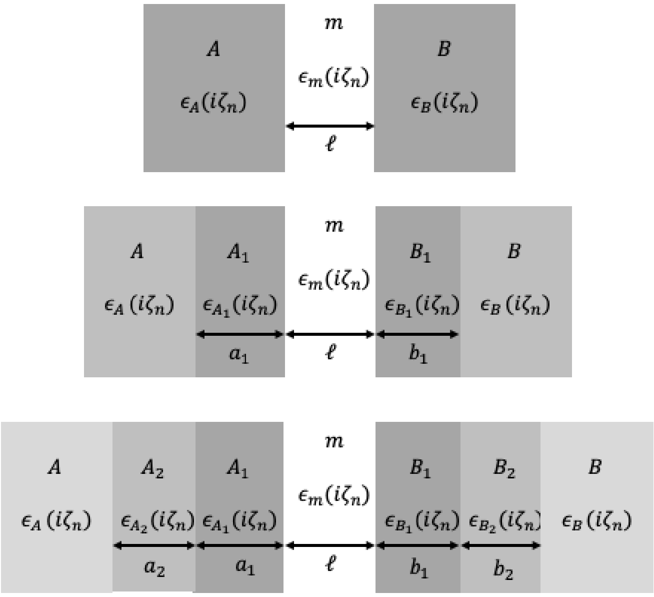

Figure S 1: Semi-infinite half spaces A and B, separated by a distance ℓ. Top: bulk materials A and B described by the complex dielectric functions $\epsilon_A(i\zeta_n)$ and $\epsilon_B(i\zeta_n)$ at the complex Matsubara frequency. Middle: double-layer materials with the additional A_1 and B_1 layers of thickness a_1 and b_1. Bottom: triple layers with two added layers, A_1 and A_2, B_1 and B_2, of the corresponding thickness a_1 and a_2, b_1 and b_2.

Using the values of the dielectric constant of ethanol ($\epsilon_{\mathrm{EtOH}} = 24.3$) and cyclohexane ($\epsilon_{\mathrm{C_6H_{12}}} = 2.02$) as the upper and lower bounds for the known solvents that could enable quantum entrapment, we consider additional mixtures that could, in principle, sustain quantum entrapment. The dielectric functions are calculated as a combination of the oscillator models describing cyclohexane and ethanol following a summation of ϵ_{EtOH} and $\epsilon_{\mathrm{C_6H_{12}}}$ with normalizing/weighted prefactors (w_1 and w_2); $\epsilon_m = w_1\epsilon_{\mathrm{EtOH}} + w_2\epsilon_{\mathrm{C_6H_{12}}}$ (see Figure S2a). Although the ordering of the dielectric functions can be seen to switch at around 18 eV, all solvents for which the interaction was calculated can be seen to possess an equilibrium separation. Figure S2b shows that by varying the dielectric function of the medium between those described by ϵ_{EtOH} and $\epsilon_{\mathrm{C_6H_{12}}}$, one can gain a soft control of the equilibrium separation with 11 possible equilibrium separations predicted between Au and a PTFE - Au interface. Given the various binary mixture models[1,2] and the vastly different dielectric nature of the considered solvents, it is reasonable to assume that quantum entrapment can occur in a wide range of solvents.

Previously, Ederth[5] also reported a strong attractive Casimir force (of the order of μN per metre) measured by SFA in air between two identical gold surfaces with a more complex double layer structure containing a hydrocarbon layer on top of gold. The two perpendicular cylinders with radius of $R_{eff} = 2$ cm were coated with a gold layer of 200 nm thickness and a thin $a_1 = 2.1$ nm overlayer of hydrocarbon (hexadecanethiolate). We have calculated the Casimir force acting between double layer Au - hydrocarbon interfaces in air (shown in Figure S3 of the Supporting Information) which is in excellent agreement with the measurements of Ederth.[5] The cylinders in this double-layer system were represented by semi-infinite gold half-spaces with a hydrocarbon layer on top, and the dielectric functions for gold and hydrocarbon were taken from references.[4,5] Generally, the Casimir force between two ideal parallel conducting interfaces is proportional to the cross-sectional area of the interfaces, and it varies with the separation distance as $F(\ell) \propto 1/\ell^4$. Apart from the dependence on the separation distance, the Casimir force includes only on the fundamental values such as

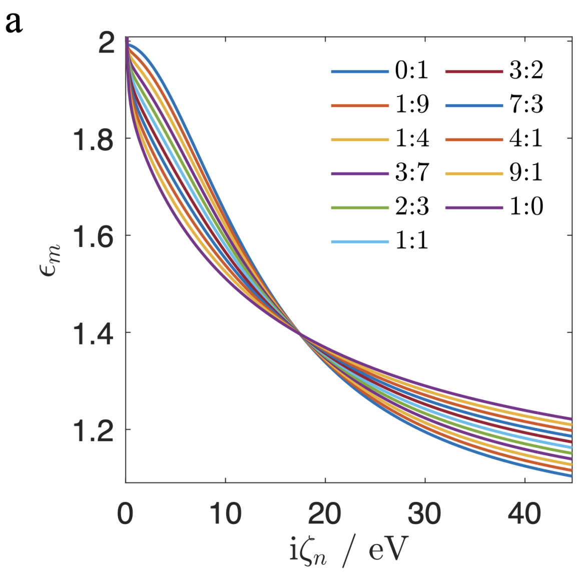

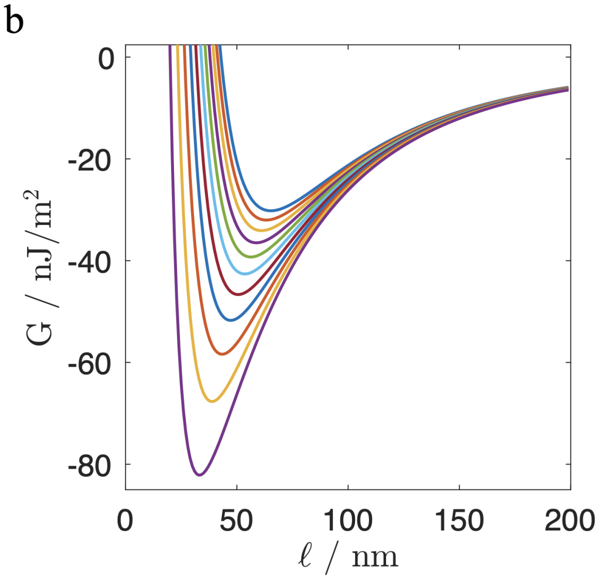

Figure S 2: (a) The dielectric functions calculated as a combination of the oscillator models describing cyclohexane and ethanol as a function of $i\zeta_n$; cyclohexane is illustrated by the dark blue line (0:1), pure ethanol is depicted by the purple line (1:0), with the intermediate cases labelled accordingly. (b) The interaction energy, per unit area, between Au surface and a PTFE - Au double layer (the thickness of PTFE a_1 = 70 nm); EtOH = 0.1 C_6H_{12} = 0.9 red, EtOH = 0.2 C_6H_{12} = 0.8 dark yellow, EtOH = 0.3 C_6H_{12} = 0.7 purple, EtOH = 0.4 C_6H_{12} = 0.6 green, EtOH = 0.5 C_6H_{12} = 0.5 light blue, EtOH = 0.6 C_6H_{12} = 0.4 maroon, EtOH = 0.7 C_6H_{12} = 0.3 dark blue, EtOH = 0.8 C_6H_{12} = 0.2 orange and EtOH = 0.9 C_6H_{12} = 0.1 yellow. PTFE and EtOH were modelled using oscillators taken from,[3] with gold modelled using an oscillator taken from.[4]

Planck's constant and the speed of light.

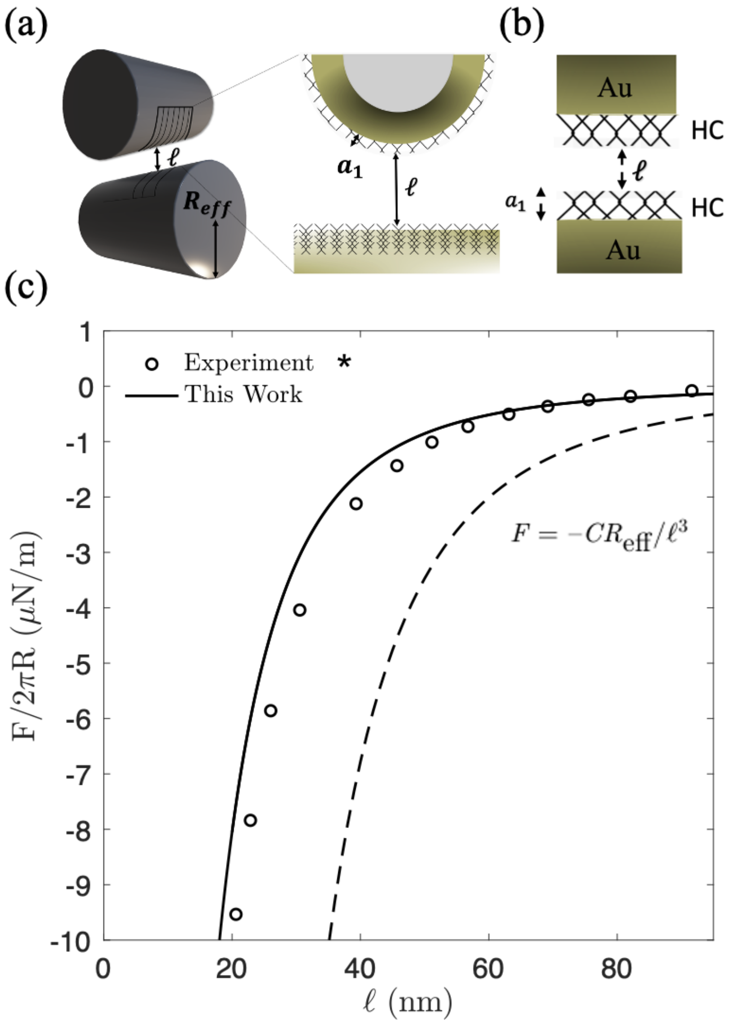

Figure S 3: The Casimir force acting between double layer Au - hydrocarbon interfaces in air. Illustration of SFA experiment (a); computational setup (b); the Casimir force as a function of the separation distance (c) showing the results of the experimental SFA measurements[5] (open circles), calculated in this work (solid line), and the case of two ideal conductors (dashed line).

References

(1) Amooey, A. A. Improved mixing rules for description of the permittivity of mixtures. *Journal of Molecular Liquids* **2013**, *180*, 31–33.

(2) Reis, J. C. R.; Iglesias, T. P.; Douhéret, G.; Davis, M. I. The permittivity of thermodynamically ideal liquid mixtures and the excess relative permittivity of binary dielectrics. *Phys. Chem. Chem. Phys.* **2009**, *11*, 3977–3986.

(3) Gudarzi, M. M.; Aboutalebi, S. H. Self-consistent dielectric functions of materials: Toward accurate computation of Casimir – van der Waals forces. *Science Advances* **2024**, *7*, eabg2272.

(4) Parsegian, V. A. *Van der Waals forces: a handbook for biologists, chemists, engineers, and physicists*; Cambridge University Press: New York, 2006.

(5) Ederth, T. Template-stripped gold surfaces with 0.4-nm rms roughness suitable for force measurements: Application to the Casimir force in the 20–100-nm range. *Phys. Rev. A* **2000**, *62*, 62104.
